# Supplementary material for: Mechanisms governing the pioneering and redistribution capabilities of the non-classical pioneer PU.1
Source: Nat Commun. 2020 Jan 21;11:402. doi: 10.1038/s41467-019-13960-2 (PMC6972792; doi:10.1038/s41467-019-13960-2)
Supplement: Supplementary file 7 — Source data [file 41467_2019_13960_MOESM7_ESM.zip › Source_Data/Figure6/Figure6B_MotifScanOutput/ETS1_PU.1/homerResults/motif32.similar.html]

motif32

## Information for motif32

T
G
A
C
C
T
G
A
A
T
G
C
A
G
T
C
G
C
T
A
A
C
T
G
C
T
A
G
C
A
T
G
C
A
T
G
T
A
C
G
G
A
T
C
T
C
A
G
  
Reverse Opposite:  

A
G
T
C
C
T
A
G
A
T
G
C
G
T
A
C
G
T
A
C
A
G
T
C
A
G
T
C
C
G
A
T
T
C
A
G
T
A
C
G
G
A
C
T
A
C
T
G
  

|  |  |
| --- | --- |
| p-value: | 1e-47 |
| log p-value: | -1.086e+02 |
| Information Content per bp: | 1.690 |
| Number of Target Sequences with motif | 624.0 |
| Percentage of Target Sequences with motif | 3.94% |
| Number of Background Sequences with motif | 543.7 |
| Percentage of Background Sequences with motif | 1.70% |
| Average Position of motif in Targets | 101.3 +/- 57.3bp |
| Average Position of motif in Background | 100.7 +/- 61.3bp |
| Strand Bias (log2 ratio + to - strand density) | 0.1 |
| Multiplicity (# of sites on avg that occur together) | 1.03 |
| Motif File: | file (matrix) reverse opposite |

### Similar de novo motifs found

|  |  |  |  |  |  |  |  |
| --- | --- | --- | --- | --- | --- | --- | --- |
| Rank | Match Score | Redundant Motif | P-value | log P-value | % of Targets | % of Background | Motif file |
| 1 | 0.965 | A G T C C A T G A T G C A G T C G T A C A G T C A T G C G C A T C T A G A T C G G A C T A C T G T C A G | 1e-44 | -101.643133 | 2.22% | 0.68% | motif file (matrix) |
| 2 | 0.913 | A G T C G T A C G T A C A G T C A G T C A C G T C T G A A T C G G C A T C T A G | 1e-34 | -78.827448 | 3.39% | 1.58% | motif file (matrix) |
| 3 | 0.852 | T A G C G T A C C T G A A T G C G A C T C T G A T C A G C T A G C T A G C A T G T C A G | 1e-22 | -51.413421 | 1.45% | 0.54% | motif file (matrix) |
